# Supplementary material for: Resolution of ranking hierarchies in directed networks
Source: PLoS One. 2018 Feb 2;13(2):e0191604. doi: 10.1371/journal.pone.0191604 (PMC5796714; doi:10.1371/journal.pone.0191604)
Supplement: S1 Appendix — (PDF) [file pone.0191604.s001.pdf]

Resolution of ranking hierarchies in directed networks

Supporting information

Elisa Letizia, Paolo Barucca, Fabrizio Lillo

January 11, 2018

Detailed proofs

In this Supporting material we present details and extended formulae for the propositions.

To start, we consider the values of agony for general d depending on the choice of the alternative rankings.

- **No inversion and splitting.** When $b < 0$, each class is divided into 2^{-b} classes. As for the affinity matrix, the only part affected by the change in the ranking is the one above the diagonal, which has no impact on the computation of $\mathbb{E}[A_d(G, r^{(b)})]$. Hence one has

$$\mathbb{E}[A_d(G, r^{(b)})] = s\left(\frac{N}{R}2^b\right)^2 \sum_{k=0}^{2^{a-b}-1} (k+1)^d (2^{a-b} - k)\,. \tag{1}$$

- **No inversion and merging.** When $b \geq 0$, for any pair (i, j) it holds:

$$\mathbb{E}[m_{ij}] = \left(\frac{N}{R}\right)^2 \begin{cases} 2^{2b}s & j < i \\ (2^b-1)p + 2^{b-1}(2^b+1)s + (2^{b-1}-1)(2^b-1)q & j = i \\ p + (2^{2b}-1)q & j = i+1 \\ 2^{2b}q & j > i+1\,, \end{cases} \tag{2}$$

  which gives

$$\begin{aligned}\mathbb{E}[A_d(G, r^{(b)})] =& s\left(\frac{N}{R}2^b\right)^2 \sum_{k=1}^{2^{a-b}-1} (k+1)^d (2^{a-b} - k) + \\ &+ 2^{a-b}\left((2^b-1)p + 2^{b-1}(2^b+1)s + (2^{b-1}-1)(2^b-1)q\right)\end{aligned}$$

- **Inversion and merging.** When $b \geq 0$ the expression for agony of the inverted ranking becomes

$$\begin{aligned}\mathbb{E}[A_d(G, r^{(i,b)})] =& 2^{2b}\left(\frac{N}{R}\right)^2 q \sum_{k=2}^{2^{a-b}-1} (k+1)^d \left(2^{a-b} - k\right) + \\ &+ 2^d\left(\frac{N}{R}\right)^2 \left(2^{a-b}-1\right)\left(\left(2^{2b}-1\right)q + p\right) + \\ &+ 2^{a-b}\left(\frac{N}{R}\right)^2 \left(\left(2^b-1\right)p + \left(2^{b-1}-1\right)\left(2^b-1\right)q + 2^{b-1}\left(2^b+1\right)s\right)\end{aligned}$$

- **Inversion and splitting** When $b < 0$

$$
\begin{aligned}
\mathbb{E}[A_d(G, r^{(i,b)})] = &\left(\frac{N}{R}2^b\right)^2 \sum_{k=0}^{2^{-b}-1} (k+1)^d \left(2^a \left(2^{-b}-k\right) s + \left(2^a-1\right) kp\right) + \\
&+ \left(\frac{N}{R}2^b\right)^2 \sum_{k=0}^{2^{-b}-1} \left(k+1+2^{-b}\right)^d \left(\left(2^a-1\right)\left(2^{-b}-k\right) p + \left(2^a-2\right) kq\right) + \\
&+ \left(\frac{N}{R}2^b\right)^2 q \sum_{k=0}^{(2^a-2)2^{-b}} \left(k+1+2^{1-b}\right)^d \left(\left(2^a-2\right)2^{-b}-k\right) .
\end{aligned}
$$

Then, we present the proofs of the propositions.

Proof of Proposition 1

We explicitly show that in the $d = 1$ case there exists critical values for s at which the planted ranking ceases to maximize hierarchy both for Twitter-like and Military-like hierarchies.

To determine the optimal number of classes we first treat b as a continuous variable and compute the derivative oh $\bar{h}_1$ with respect to it. The unique critical point is denoted by b^* and it is given by

$$
b^* = \frac{1}{2}\log_2 \frac{2^{2a}s + 6(q-p)}{3q-s} .
$$

Note that it must hold

$$
0 \le b \le a
$$

and we want to avoid the continuous relaxation at the boundaries so we consider the extreme values separately.

When $p \ge q > s$ (*Twitter-like hierarchy*), we first notice that

$$
\frac{\partial \bar{h}_1}{\partial b}|_{b=b*} < 0
$$

Moreover, it holds

$$
\bar{h}_1(b = a-1) > \bar{h}_1(b = a) ,
$$

that is the trivial ranking is never better than that with two classes.

Moreover, we denote with s_2 the value of s such that the rankings with two and three classes have the same value of hierarchy, i.e.

$$
\bar{h}_1 \left(b = a - \log_2 3\right) = \bar{h}_1(b = a-1) ,
$$

since for any fixed $b > 0$, $\bar{h}_1$ is monotone decreasing with respect to s,

$$
\bar{h}_1 \left(b = a - \log_2 3\right) < \bar{h}_1(b = a-1) \, \forall s \ge s_2 .
$$

Similarly, one can find the critical value s_m such that the ranking with of $R-1$ classes shares the value of hierarchy with the planted one,

$$
\bar{h}_1(b = 0) = \bar{h}_1 \left(b = a - \log_2 \left(2^a - 1\right)\right) .
$$

Finally, we can combine the results to obtain the optimal number of classes for the direct ranking in the region $p \ge q > s$:

$$
\tilde{R}^* = \begin{cases} R & s \le s_m \\ 2^{a-b^*} & s_m < s < s_2 \\ 2 & s \ge s_2 , \end{cases} \tag{3}
$$

where

$$s_m = \frac{6\,(2^a-1)\,p - 3\,(2^a-2)\,q}{2^a - 4^a + 8^a} \tag{4}$$
$$s_2 = \frac{3}{7}\frac{(4^a-12)\,q + 12p}{4^a}$$

With a reasoning similar to the one carried before, one gets that when $p \geq q > s$ the optimal number of classes for the inverted ranking is such that

$$1 \leq \tilde{R}^* \leq 2$$

hence,

$$h_1^{i,*} \leq 0,\, \forall\, p \geq q > s,\, \forall\, a\,.$$

One can conclude that the optimal ranking for the twitter-like hierarchy is the direct one with a number of classes which depends on s, according to (**??**).

When $q = 0$ (*Military-like hierarchy*), when it is defined, we have

$$\frac{\partial^2 \bar{h}_1}{\partial b^2}|_{b=b*} > 0\,,$$

so, to obtain the optimal directed ranking we only need to check the extreme values for b, i.e. $b = 0$, $b = a$. The optimal number of classes for the direct ranking is given by

$$\tilde{R}^* = \begin{cases} R & s \leq s_{m|_{q=0}} \\ 1 & \text{otherwise}\,, \end{cases}$$

where

$$s_1 = \frac{6p}{2^a(1+2^a)}\,.$$

Then, one can consider the inverted ranking.

It easy to verify that

$$\mathbb{E}[A_1(G, r^{(i,b)})] > \mathbb{E}[A_1(G, r^{(p)})],\, \forall\, b < 0\,,$$

that is, also for the inverted ranking splitting is never optimal on average.

As for merging, the optimal choice for b is given by

$$b^{i,*} = \frac{1}{2}log_2\frac{2p}{s}\,,$$

which is well defined when $s > \frac{2}{4^a}p$ and satisfies $\frac{a}{2} \leq b^{i,*} \leq a$. The optimal number of classes fro the inverted ranking is given by

$$\tilde{R}^{i,*} = \begin{cases} 1 & s \leq s_2^i \\ 2 & s_2^i < s \leq s_3^i \\ 2^{a-b^{i,*}} & s > s_3^i \end{cases}\,,$$

where

$$s_2^i = 2^{2-2a}p$$
$$s_3^i = 3s_2^i\,.$$

When $s \leq s_1$, the planted ranking is optimal and non zero and decreasing, and

$$s_2^i < s_1 < s_3^i\,. \tag{5}$$

Denote by s_i the value of s such that

$$\bar{h}_1^i(b = a - 1) = \bar{h}_1(b = 0)\,.$$

One gets

$$s_i = \frac{12p}{3\ 2^a + 2^{2a+1} - 2}\,,$$

and when $s > s_i$ the optimal inverted ranking has a higher value of hierarchy than the planted, which is the optimal directed one.

Finally, one can write the expression for the estimate of the optimal value of h in proposition **??**.

For $p \geq q > s$,

$$\bar{h}_1^* = \begin{cases} -\frac{(2^a-2)(-6(2^a-1)q+2^a(2^a+2)s-6p)}{6(2^a(2p-3q+s)+4^a(q+s)-2p+2q)} & s \leq s_m \\ \frac{3((4^a+2)q-2p)\sqrt{\frac{4^a s-6p+6q}{3q-s}}-2^{a+1}(4^a s-6p+6q)}{3\sqrt{\frac{4^a s-6p+6q}{3q-s}}(2^a(2p-3q+s)+4^a(q+s)-2p+2q)} & s_m < s < s_2 \\ \frac{4^a(q-s)+4p-4q}{2(2^a(2p-3q+s)+4^a(q+s)-2p+2q)} & s \geq s_2\,. \end{cases}$$

When $q = 0$,

$$\bar{h}_1^* = \begin{cases} \frac{2^a(6p+s)-8^a s-6p}{6(2^a-1)p+3\ 2^a(2^a+1)s} & s \leq s_i \\ \frac{4^a s-4p}{2(2^a(2p+s)+4^a s-2p)} & s_i < s \leq s_3^i \\ \frac{-2^{a+\frac{3}{2}} s\sqrt{\frac{p}{s}}+4^a s+2p}{2^a(2p+s)+4^a s-2p} & s > s_3^i\,. \end{cases}$$

Proof of Proposition 2

We here proceed to show that in the $d = 0$ case (FAS), both for Twitter-like and Military-like hierarchies, agony is minimized by the ranking where nodes are partitioned in singletons. When $b > 0$, the derivative of h with respect to b is negative hence the planted ranking is better that any other with a fewer number of classes. Instead, when $b < 0$ one has

$$\mathbb{E}[A_0(G, r^{(b)})] = s(2^a + 2^b)\left(\frac{N}{R}\right)^2$$

which implies

$$\mathbb{E}[A_0(G, r^{(b)})] < \mathbb{E}[A_0(G, r^{(p)})],\ \forall\, b < 0\,,$$

and

$$\frac{\partial \bar{h}_0}{\partial b} = -\frac{2^{a+b-1}}{m} s < 0 \quad \forall\, b < 0$$

So the optimal ranking is obtained for the limit value of b

$$b^* = -\log_2 \frac{N}{R}, \quad \tilde{R}^* = N\,.$$

Similar computations give that any inverted ranking (i.e $\forall\, b$) has never a higher value of hierarchy than the the ranking we just discussed.

One get the formula in proposition 2

$$h_0^* = 1 - \frac{2^{2a}(N+1)s}{(2^{2a}(q+s) + 2^a(2p - 3q + s) - 2p + 2q)\,N}$$

Proof of Proposition 3

For the case $d = 2$ one can follow the same procedure we showed for $d = 1$ and find the critical values for resolution threshold.

When $p \geq q > s$, the optimal number of classes is given by

$$\tilde{R}_2^* = \begin{cases} R & s \leq s_{2,m} \\ 2^{a-b_2^*} & s_{2,m} \leq s \leq s_{2,1} \\ 1 & s \geq s_{2,1} \,, \end{cases}$$

where

$$\begin{aligned} b_2^* =& \log_2 \Big(\frac{2\sqrt[3]{2}\left(2^{2a}s - 3p + 3q\right)}{\sqrt[3]{\beta + 3^5\ 2^{3a}q^2 s - 3^4\ 2^{3a+2}qs^2 + 3^3\ 2^{3a+2}s^3}} + \\ &+ \frac{\sqrt[3]{\frac{1}{3}\beta + 2^4\ 2^{3a}q^2 s - 3^3\ 2^{3a+2}qs^2 + 3^2\ 2^{3a+2}s^3}}{\sqrt[3]{2}\,3^2(3q-2s)} \Big) \,, \\ \beta =& \sqrt{3^6\ 2^{6a}s^2(3q-2s)^4 - 2^5\ 3^3(3q-2s)^3\left(4^a s - 3p + 3q\right)^3} \,. \end{aligned} \tag{6}$$

is the unique zero of the first order derivative of $\bar{h}_2$ with respect to b, and

$$\begin{aligned} s_{2,m} &= \frac{6\left(2^{1-a}(q-p) + 2p - q\right)}{-3\ 2^a + 2^{3a+1} + 4^a + 4} \\ s_{2,1} &= \frac{2^{2a}q + 4p - 4q}{3\,2^{2a}} \end{aligned}$$

with $s_{2,1}$ being the value of s such that

$$\bar{h}_2(b = a-1) = \bar{h}_2(b = a) = 0 \,.$$

When $q = 0$, the planted ranking is optimal and gives positive $\bar{h}_2$ when $s < s_{2,1}^0$, where

$$s_{2,1}^0 = \frac{3\ 2^{2-a}p}{5\ 2^a + 4^a + 4} \,.$$

For the inverted ranking instead one can compute the optimal choice for the number of classes, that is

$$\tilde{R}_2^{i,*} = \begin{cases} a & s \leq s_{2,2}^i \\ a-1 & s_{2,2}^i p < s < s_{2,3}^i \\ \frac{\log\left(\frac{6p}{s}\right)}{\log(4)} & s > s_{2,3}^i \,, \end{cases}$$

where

$$b_2^{i,*} = \frac{\log\left(\frac{6p}{s}\right)}{2\log(2)} \,,$$

and

$$\begin{aligned} s_{2,2}^i &= \frac{12}{2^{2a}}p \\ s_{2,3}^i &= 3s_{2,2}^i \,. \end{aligned}$$

For any choice of p and a, it holds

$$s_{2,1} < s_{2,2}^i \,,$$

so the inverted ranking is optimal for $s > s_{2,2}^i$.
