# Supplementary material for: Resolution of ranking hierarchies in directed networks
Source: PLoS One. 2018 Feb 2;13(2):e0191604. doi: 10.1371/journal.pone.0191604 (PMC5796714; doi:10.1371/journal.pone.0191604)
Supplement: S2 Table — (PDF) [file pone.0191604.s003.pdf]

Resolution of ranking hierarchies in directed networks
S3 Table

Elisa Letizia, Paolo Barucca, Fabrizio Lillo

January 11, 2018

Numerical results

For each network and for each class, the table contains the size of the class, n_i, as a percentage of the total number of nodes), the value of h, and the number of sub-classes inferred (R).

Table 1: **Real networks: details for classes**

	Wikivote			HiggsReply			HiggsMention			Amazon		
cl.	$n_i(\%)$	h^*	R	$n_i(\%)$	h^*	R	$n_i(\%)$	h^*	R	$n_i(\%)$	h^*	R
1	0.67	1*	1	0.60	0.03	2	0.77	0.13	2	0.02	< 0.01	3
2	0.01	0	1	0.31	0.34	2	0.16	0.78	4	0.03	0.01	3
3	< 0.01	0.24	3	0.04	0.64	2	0.03	0.78	3	0.10	0.01	3
4	0.01	0.38	6	0.01	1	2	0.01	0.80	2	0.20	0.01	5
5	0.02	0.26	5	< 0.01	0.50	2	< 0.01	0.81	2	0.25	0.05	6
6	0.04	0.23	6	< 0.01	1	2	< 0.01	0.85	2	0.20	0.08	6
7	0.06	0.20	5	< 0.01	1	3	< 0.01	0.53	2	0.11	0.08	6
8	0.09	0.32	8	< 0.01	0.67	5	< 0.01	0.65	3	0.06	0.07	5
9	0.08	0.72	10	0.01	0.24	2	< 0.01	0.66	4	0.02	0.06	5
10	0.04	1	2	< 0.01	0.83	2	< 0.01	0.55	4	0.01	0.05	5
11	< 0.01	0	1	< 0.01	1*	1	< 0.01	0.59	7	< 0.01	0.06	4
12	< 0.01	1*	1	< 0.01	1*	1	< 0.01	0.46	6	< 0.01	0.05	4
13				< 0.01	1*	1	< 0.01	0.60	6	< 0.01	0.04	3
14							< 0.01	0.66	6	< 0.01	0.05	4
15							< 0.01	0.82	1	< 0.01	0.07	3
16							< 0.01	0.69	1	< 0.01	0.05	3
17							< 0.01	1*	1	< 0.01	0	1
18							< 0.01	1*	1			
19							< 0.01	1*	1			
20							< 0.01	1*	1			

* empty
